# The opioid crisis: need for systems science research

**DOI:** 10.1186/s12961-020-00598-6

**Published:** 2020-08-08

**Authors:** Mohammad S. Jalali, Michael Botticelli, Rachael C. Hwang, Howard K. Koh, R. Kathryn McHugh

**Affiliations:** 1 MGH Institute for Technology Assessment, Harvard Medical School, 101 Merrimac St, Suite 1010, Boston, MA 02114 United States of America; 2 MIT Sloan School of Management, 100 Main St, Cambridge, MA 02142 United States of America; 3 Grayken Center for Addiction, Boston Medical Center, Boston, MA United States of America; 4 T.H. Chan School of Public Health, Harvard University, Boston, MA United States of America; 5 Harvard Kennedy School, Harvard University, Cambridge, MA United States of America; 6 Division of Alcohol and Drug Abuse, McLean Hospital, Belmont, MA United States of America

**Keywords:** opioids, opioid use disorder, systems science, simulation modelling

## Abstract

The opioid epidemic in the United States has had a devastating impact on millions of people as well as on their families and communities. The increased prevalence of opioid misuse, use disorder and overdose in recent years has highlighted the need for improved public health approaches for reducing the tremendous harms of this illness. In this paper, we explain and call for the need for more systems science approaches, which can uncover the complexities of the opioid crisis, and help evaluate, analyse and forecast the effectiveness of ongoing and new policy interventions. Similar to how a stream of systems science research helped policy development in infectious diseases and obesity, more systems science research is needed in opioids.

## Background

The staggering increase in opioid misuse, opioid use disorder diagnoses and overdose fatalities in the past two decades has yielded an urgent need in understanding the most effective public health interventions for addressing opioid-related harms. The understanding that the opioid crisis is not solely a behavioural and/or a biological problem, but is also a problem influenced by many moving parts in the broader social-ecological system [1] indicates the importance of systems science tools to help assess the interconnections of the risk factors. Despite the increased attention to opioids, there are still two major shortcomings in the literature, namely (1) a lack of understanding of the complexity of the system, e.g. the interconnections of the risk factors at different levels, and (2) a tremendous lack of evidence to inform policy-making. Moreover, data sources are often not integrated across agencies or across federal, state and local levels.

As the opioid crisis is a multi-faceted issue [1], analysing the interconnections among the fragments of the systems can inform policy development and public health decisions. Currently, the most common approach to research is that the large system is broken down into its components and they are studied individually. Also critical, and often missing, is identifying the explanatory pathways and potential interactions among the components of the system.

Furthermore, the complexity of this crisis requires consideration of the unintended consequences of any policy actions. However, in complex systems, even experts fail to fully understand the unintended consequences of their decisions, particularly over the long-term [2–6]; consequently, proposed solutions may introduce new problems or fail to produce lasting results. This issue is often attributable to observing and analysing a fragment of the system (only seeing the top of the iceberg) or to a lack of understanding of the interconnections among system components. Take, for example, efforts to reduce access to prescription opioids. Although supply-side interventions, such as the expansion of prescription drug monitoring programmes and improved provider education, have already shown benefits, such interventions may not substantively help people who have already developed opioid use disorder (OUD) and are physically dependent on opioids. For this group, failure to obtain opioids results in a highly aversive withdrawal syndrome that can motivate the escalation of use, a transition to heroin or lead to the introduction of riskier methods of administration (e.g. intravenous use). For this group, concurrent efforts to increase access to treatment and specifically reduce the barriers to effective medication for OUD is essential. Furthermore, the unintended consequences of supply-side interventions have been observed. The Centers for Disease Control and Prevention published guidelines for prescribing opioids for pain in 2016, with the aim of reducing the unnecessary or contraindicated prescription of opioids. Although this guidance was an important policy step, the authors of the original report published an update on its guidance for opioid prescribing after reported misapplications of the guidelines that had potential to harm patients [7] such as rapidly discontinuing opioid prescriptions. In general, unintended consequences have been common in addressing complex social and public health problems. For instance, the war on drugs, which resulted in mass incarceration in the United States while prisons remained in unsafe conditions with inadequate HIV/AIDS prevention and treatment measures, led to the growth in HIV/AIDS rates [8] (see [8] for several more unintended consequences resulted from the war on drugs).

The complexity of these trade-offs, which may result in different consequences in the short and long term, requires tools that can simultaneously consider these interrelationships over time. We discuss how systems science tools can help fill these gaps and integrate available data sources to better understand the ‘big picture’ and enhance policy analysis. Building upon other recent systems-based models that have been presented [9–12], we take a wide-ranging view of the multi-level complex factors that may play a role in the opioid crisis. We explain and call for the need for more systems science approaches in opioid research.

### Systems science

Systems science is a broad and interdisciplinary class of analytical and simulation modelling approaches to uncover the complexities and dynamic behaviour of a system. Using systems science allows us to understand the complex connections between the behaviour of the system (herein the number of overdose deaths that we can see and measure) and its structure (i.e. the invisible web of interconnections among risks factors affecting opioid misuse). It also provides a way to conduct trade-off analysis, especially when many policy decisions require balancing competing values (e.g. decreasing opioid supply and adequately managing pain). Systems science provides methods to distinguish and understand the multiple interactions embedded in the crisis. Additionally, a systems approach encourages the adoption of collaborative partnerships and the desertion of the traditionalist top-down and command-centric management methods to fill the knowledge gaps of the opioid crisis. Next, we discuss simulation modelling as the main systems science tool to achieve these goals and call for more simulation modelling research on opioid misuse.

### Simulation modelling and analysis

Systems science tools can be employed to develop models for simulating and analysing the behaviour of the system. They can help analyse the actual impact of the policies and identify potential unexpected consequences. In particular, the health outcomes and cost-effectiveness of alternative policies for prevention and treatment can be measured. Given that public health resources are always limited, these models are especially helpful for resource allocation – how to allocate resources to prevention or treatment strategies that optimise outcomes and are cost-effective.

Additionally, although data is currently available to some limited extent (e.g. the numbers of dispensed prescriptions and fatal overdoses reported by the Centers for Disease Control and Prevention), there is a lack of data for essential mechanisms (see [13] for more information about data challenges in opioids research). Through calibrating a model to the available data, unknown variables of the model can be estimated, which helps understand the behaviour of previously unknown mechanisms. If limited data are available with high uncertainties, simulation modelling can inform policy development through sensitivity analysis.

A major benefit of simulation models is to conduct sensitivity analysis – changing a variable in the model (e.g. prescription rate for high dose opioids) and monitoring the outcome of interest over time (e.g. the number of overdoses). This helps not only to estimate the short- and long-term effects of policy options, but also to analyse the trade-offs of benefits and risks and to pinpoint leverage points for the most effective policies. Through sensitivity analysis, the application of simulation models goes beyond the analysis of alternative strategies (policy evaluation) and can suggest opportunities for developing new policies (policy design). For instance, this type of analysis can address questions such as, for example, how would supporting (or restricting) policies for abuse-deterrent opioids impact opioid misuse and the transition dynamics to heroin? How would new policies on ultra-high dose opioids impact the trajectories of developing OUD and the trends of overdose death? How would the effects of these policies vary across patients in different age groups and genders?

The use of systems science also goes beyond prediction to uncover the complexities of the system and understand the root causes of the problems. Some behavioural questions to analyse could be, for example, how would social contagion (e.g. through connections with people who use or misuse opioids) impact the exposure of opioid-naive individuals to opioids? How would the changes in the rates of overdose affect physicians’ perception of opioid safety, and how would their perception impact their prescribing behaviour and, accordingly, the number of overdoses in the long term (closing the loop)? Mechanisms to answer these and many other social questions are often absent in statistical and epidemiological studies due to the lack of quantitative data. In the complete absence of such data, qualitative systems methods (e.g. causal loop diagrams and subsystem diagrams) can enhance our intuitions and overcome the impediments to learning the complexity of the system (see [14] for more discussion). In fact, system science tools have assisted learning in many complex social problems that key variables cannot be readily quantify or optimise [15, 16].

Overall, simulation modelling helps answer ‘what-if’ policy questions and expand and refine the mental models of policy-makers. Furthermore, from the perspective of policy-makers, no one solution can solve this public health problem (e.g. short-term harm reduction interventions for individuals with OUD may not be fully effective without follow-up for the treatment of OUD). To address this concern, simulation modelling can be employed to provide insights on what combinations of interventions, policies and resource allocation strategies can best achieve the desired goals. Hence, simulation models can replace the short-term, narrow and static understanding of the problem with a long-run, broad and flexible outlook fit to address the public health crisis. See Figure A1 in the Additional File 1 for the general steps for developing a simulation model.

Despite the benefits, such as accounting for complex relationships within the system and providing a clear way to evaluate potential policies, simulation models are no panacea and their use is limited and subject to careful considerations. The complex web of factors influencing opioid misuse makes it hard to develop system models. This is essentially bold in opioid misuse research given that, for many risk factors (e.g. who will escalate from use to misuse to use disorder), sufficient data is not available and, often, their underlying causes are not well known, leading to reliance on data assumptions that may or may not be accurate. Moreover, there is often a lack of knowing if a policy would provide statistically significant change. Hence, models remain highly sensitive to the expertise and understanding of the modelers and domain experts. Subsequently, no systems model for opioid misuse will ever be fully accurate but, if they are evidence-based and carefully assessed, they can be used for the benefits discussed earlier. Particularly, simulation models that are grounded in the literature and available data, reported with a clear presentation of all assumptions, presented with detailed sensitivity analyses on assumptions, and developed with a minimum number of assumptions are needed – a model based on a mountain of assumptions would be more harmful than helpful.

Hence, future research should employ system science tools and study the dynamic interconnections of the major factors in opioid misuse to develop effective interventions and policies.

### Moving forward to address the opioid crisis

Systems science and simulation modelling have been widely used in public health [17–19]; however, their applications have been limited in opioid research. There have recently been modelling-based studies on opioids focusing on educational interventions [9], tamper-resistant formulations and informal sharing of opioids [10], interventions targeting prescription opioid misuse [11], and health outcomes of several prevention and treatment policies [12]. See [20] for more examples of simulation models in opioids research and discussions about the strengths and limitations of their methods. These models make important contributions; however, they have limitations in their scope, so there remains a need for more models to unveil the complexities of the crisis.

In fact, no one simulation model for opioids can answer all research questions. In other areas of research, such as obesity [21, 22] and infectious diseases (e.g. HIV/AIDS) [23–25], a stream of systems science research helped intervention and policy development. For instance, the National Institutes of Health’s Models of Infectious Disease Agent Study programme, started in 2004, created a network of over 20 universities to develop infectious disease epidemic models, which has resulted in impactful strategies to prevent infectious diseases or minimise their impact. There are also over 200 simulation-modelling reports in the literature informing HIV treatment decisions that serve critical roles in informing HIV-related policies and in evaluating the outcomes and cost-effectiveness of specific interventions [26, 27]. A similar investment in systems science research is needed to inform policy development in opioids.

## Conclusion

The dynamic environment in which the opioid crisis is embedded indicates the importance of utilising systems science research (e.g. qualitative system maps and quantitative simulation tools) that provides information on system diagnoses and performance changes of the crisis. Using these tools, researchers can develop models to measure and analyse the interconnections among the risk factors to better understand the complexities of the crisis. Critically, these models can also be used to conduct trade-off analysis and evaluate the effectiveness of ongoing (and new) policy efforts to provide evidence to inform where to focus efforts and allocate limited resources. Overall, these tools effectively examine and can help experts comprehend the underlying complexities and interconnections of variables to better establish effective prevention and treatment solutions.

While the interest in the application of systems science in public health and health policy has been increasingly growing [28], application to opioid research has been slow. The shortage of effective models for addressing the opioid crisis indicates an urgent need for systems science tools, and we encourage researchers and experts in systems science and opioids to develop and utilise systems science tools to address the crisis, which will require intensive interdisciplinary research and more attention and support from state and federal agencies.

## Supplementary information

**Additional file 1.** Steps of the modelling process; Figure A1.

## Data Availability

Not applicable.

## Abbreviation

OUD: opioid use disorder
