# Supplementary material for: The opioid crisis: need for systems science research
Source: Health Res Policy Syst. 2020 Aug 8;18:88. doi: 10.1186/s12961-020-00598-6 (PMC7414582; doi:10.1186/s12961-020-00598-6)
Supplement: Supplementary file 1 — Additional file 1. Steps of the modelling process; Figure A1. [file 12961_2020_598_MOESM1_ESM.docx]

# *Additional file for*

**The Opioid Crisis: Need for Systems Science Research**

## **Steps of the modeling process**

Figure A1 presents the steps of the modeling process. This process is not a linear sequence of steps and is iterative. One needs to start from step one (problem articulation) and follow the cycle but the results of any step can increase the understanding of prior steps; accordingly, any earlier step can be revised. See [1] for more information about each step and guidelines to develop a proper model.

**Iterative process:** Results of any stage can yield insights to revise any ‘*earlier’* step.

Figure A1: Modeling process (adapted from [1])

**References**:

1. Sterman, J.D., *Learning from evidence in a complex world.* Am J Public Health, 2006. **96**(3): p. 505-14.
